# Data on cadmium removal from synthetic aqueous solution using garbage ash

**DOI:** 10.1016/j.dib.2018.08.163

**Published:** 2018-09-01

**Authors:** Mehdi Qasemi, Ahmad Zarei, Mojtaba Afsharnia, Rezvan Salehi, Mohadeseh Allahdadi, Mansoureh Farhang

**Affiliations:** a Department of Environmental Health Engineering, School of Health, Gonabad University of Medical Sciences, Gonabad, Iran; b Health and Treatment Center of Al-Zahra, Gonabad University of Medical Sciences, Gonabad, Iran

**Keywords:** Cadmium, Adsorption, Garbage ash, Aqueous solution

## Abstract

This data article investigates cadmium removal efficiency using garbage ash as a cheap and effective adsorbent. Influence of different parameters, such as initial cadmium (II) concentration (mg/L), contact time (min), adsorbent dose (gr/L), pH and temperature (°C) were investigated. The characterization data of the garbage ash was determined using SEM analysis. The experimental data indicated that the adsorption of cadmium on garbage ash follows pseudo second order model and Langmuir isotherm model with *R*^2^ = 0.99. Also, the maximum adsorption capacity of adsorbent was 100.25 mg/g. Thermodynamic data showed that cadmium adsorption on garbage ash was a spontaneous and endothermic process. Based on acquired data, garbage ash could be proposed as an efficient and low-cost adsorbent for the removal of cadmium from aqueous solution.

**Specifications Table**

**Table:** 

Subject area	Chemical Engineering
More specific subject area	Adsorption
Type of data	Table, figure
How data was acquired	The uptake of cadmium (II) by the adsorbent (qe) was determined based on the subtraction of the initial and final concentration of adsorbate
	Atomic Absorption Spectrophotometer (Shimadzu, AA-7000) was used for determination of cadmium (II) concentration
Data format	Raw, analyzed
Experimental factors	For the preparation of adsorbent, garbage was placed in a furnace at 550 °C for 4.5 h to produce ash
Experimental features	Cadmium (II) adsorption from aqueous solution using garbage ash
Data source location	Gonabad, Khorasan Razavi province, Iran
Data accessibility	Data are included in this article.

**Value of the data**

• The application of adsorbent of garbage ash due to cost-effectiveness and good potential is a suitable option for the removal of Cd^2+^ from aqueous solution.
• The isotherm, thermodynamic and kinetic data will be useful for predicting the adsorption capacity, modeling and mechanism of Cd^2+^removal by garbage ash.
• These data can be important for removal of Cd^2+^ from aqueous solution.

## 1 Data

**Fig. 1:**
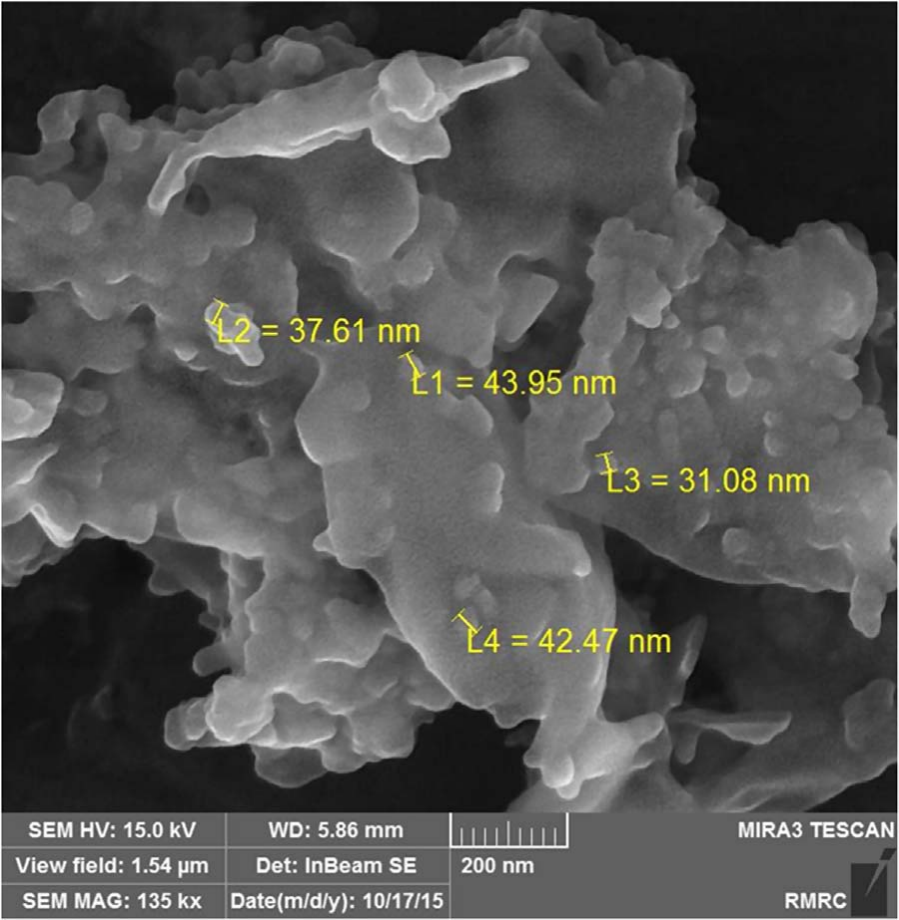
SEM micrograph of garbage ash.

**Fig. 2:**
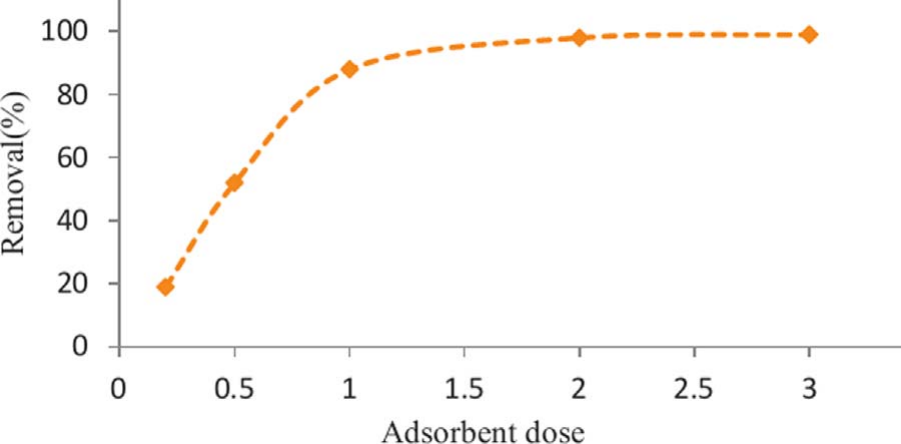
Effect of adsorbent dosage on removal efficiency (Cd^2+^ concentration: 100 mg/L, contact time: 60 min and pH: 6).

**Fig. 3:**
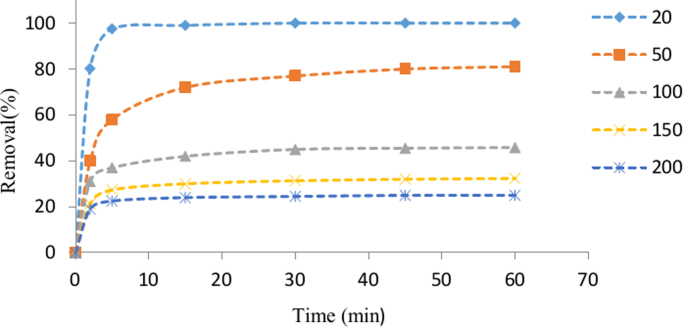
Effect of initial Cd^2+^ concentration on removal efficiency (adsorbent dosage: 0.5 g/L and pH: 6).

**Fig. 4:**
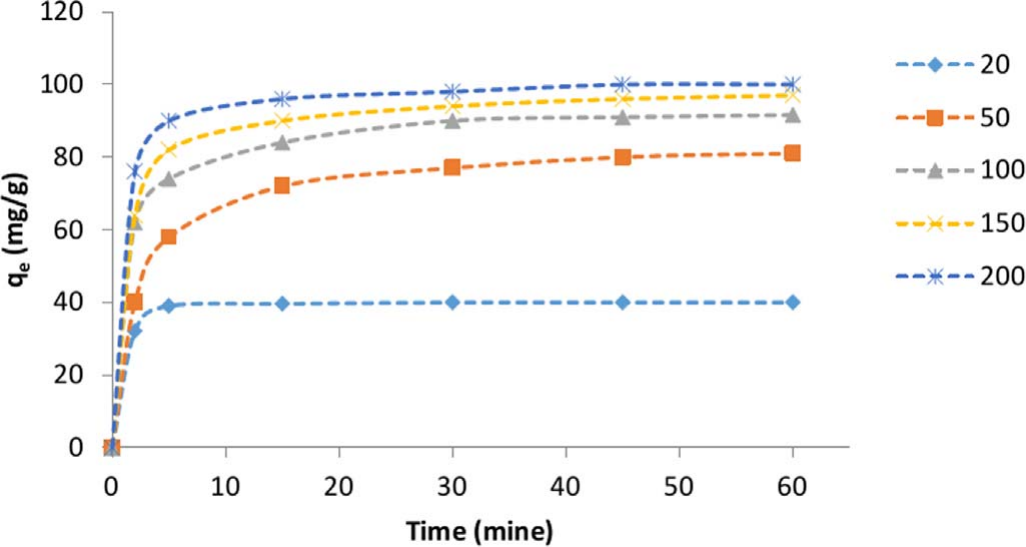
Effect of initial Cd^2+^ concentration on adsorption capacity (adsorbent dosage: 0.5 g/L and pH: 6).

**Fig. 5:**
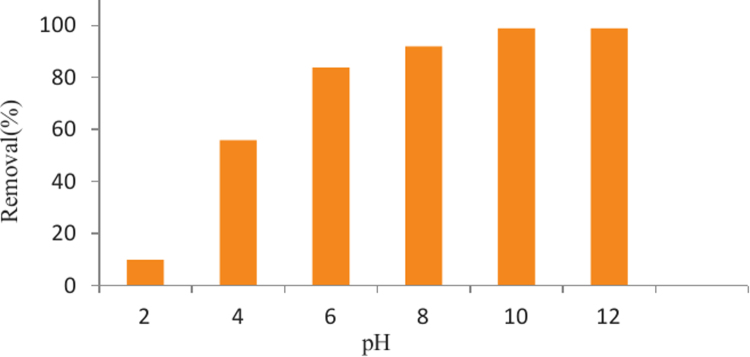
Effect of pH on Cd^2+^ removal efficiency (adsorbent dosage: 0.5 g/L, contact time: 60 min and Cd^2+^ concentration: 50 mg/L).

**Fig. 6:**
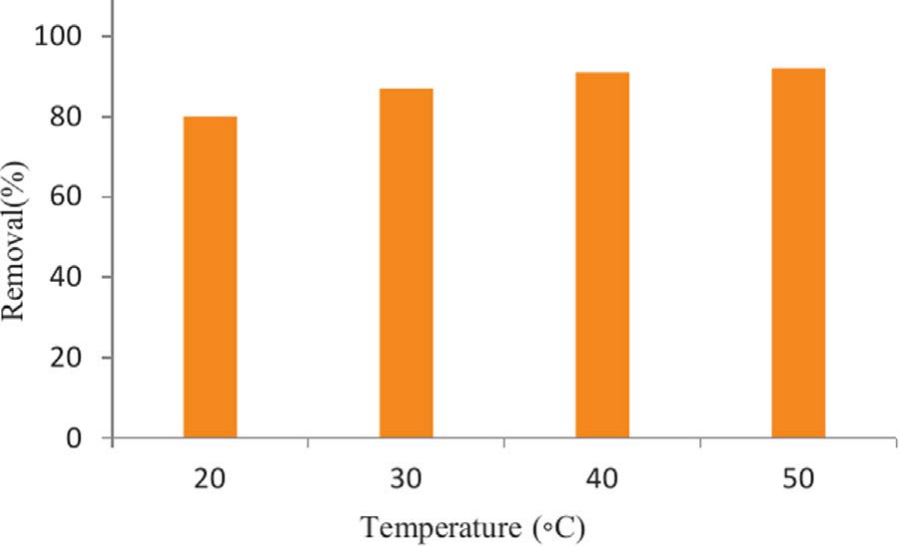
Effect of temperature on Cd^2+^ removal efficiency (adsorbent dosage: 0.5 g/L, contact time: 60 min and Cd^2+^concentration: 50 mg/L).

**Fig. 7:**
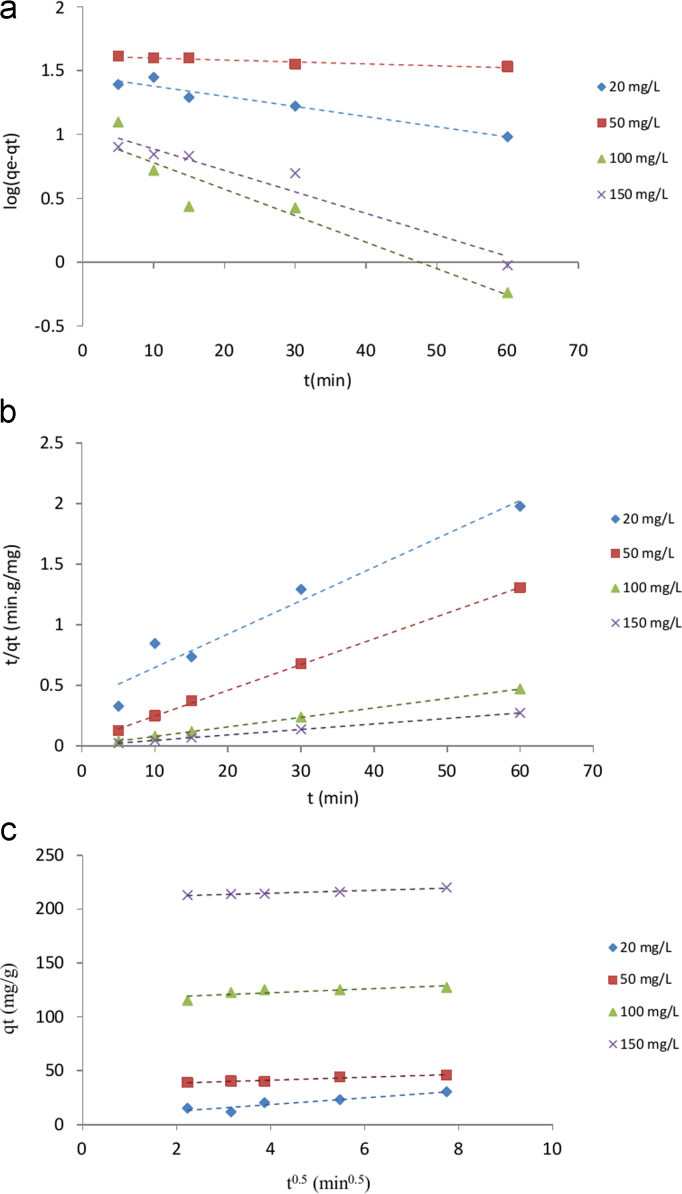
Plots of kinetic models: pseudofirstorder (a), pseudosecondorder (b) and intraparticle diffusion (C).

**Fig. 8:**
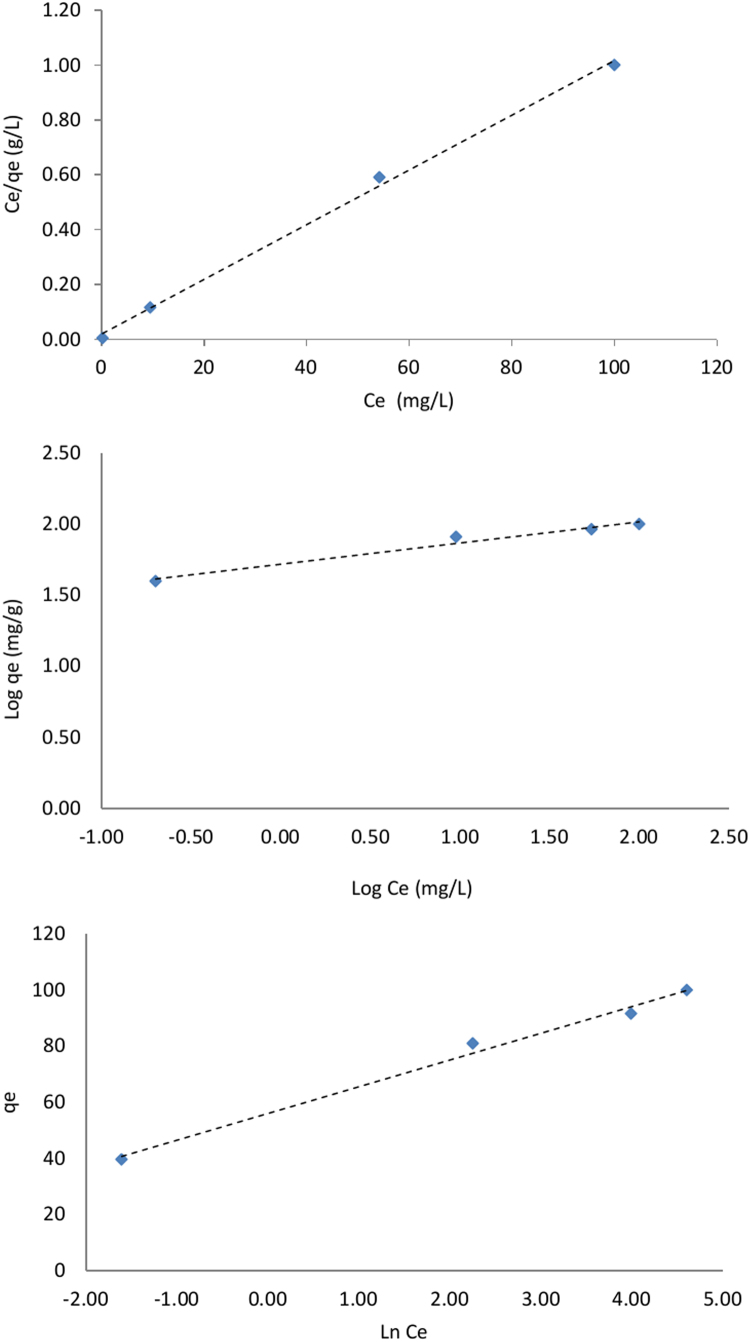
Plots of Langmuir, Freundlich, Temkin isotherms for the adsorption of Cd^2+^ by garbage ash.

**Fig. 9:**
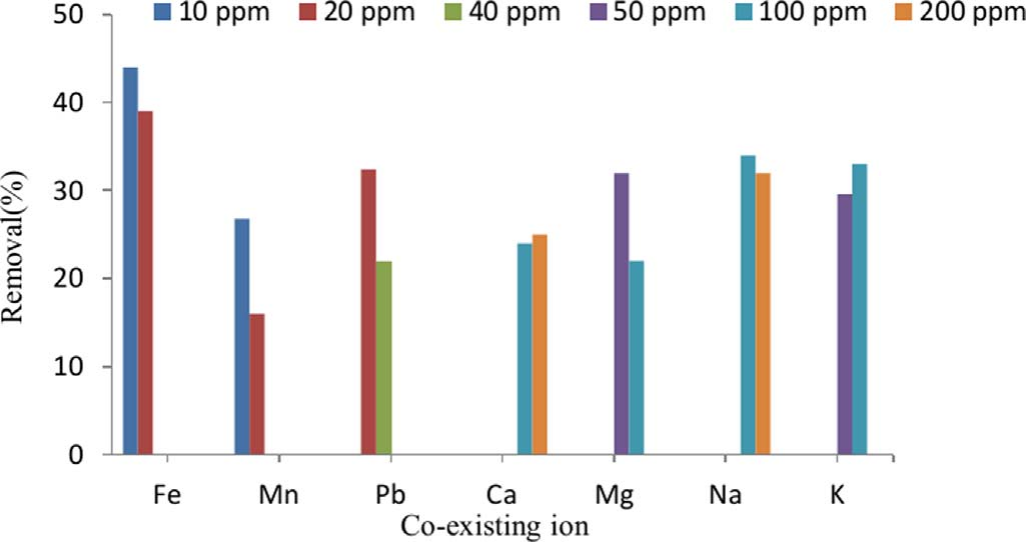
The effect of coexisting ions on Cd^2+^ removal efficiency. (adsorbent dosage: 0.5 g/L, contact time: 60 min, pH: 6 and Cd^2+^ concentration: 50 mg/L).

The SEM image of garbage ash is shown in Fig. 1. The effect of adsorbent dosage on the removal efficiency of Cd^2+^ is presented in Fig. 2. Also, Fig. 3, Fig. 4 depict the effect of initial Cd^2+^concentration on the removal efficiency and adsorption capacity. The effect of pH on Cd^2+^ removal efficiency is shown in Fig. 5. The effect of temperature on Cd^2+^ removal efficiency is also depicted in Fig. 6. The effect of coexisting ions on Cd^2+^ removal efficiency under optimized conditions is shown in Fig. 9. The plots of the kinetics and adsorption isotherms are shown in Fig. 7, Fig. 8. The kinetic equations are listed in Table 1. Kinetic parameters and correlation coefficient for Cd^2+^ adsorption by garbage ash are given in Table 2. Equations and parameters related to adsorption isotherms are summarized in Table 3. Thermodynamic parameters for Cd^2+^ removal by garbage ash are given in Table 4.

**Table 1:** Adsorption kinetics for Cd^2+^ removal by garbage ash.

Kinetic model	Formula	Plot
Pseudo first order kinetic model	$\mathrm{Log}\left(q_{e}-q_{t}\right)=\log q_{e}-\frac{k_{1}}{2.303}.t$	log(*q_e_*− *q_t_*) vs. *t*
Pseudo second order kinetic model	$\frac{t}{q_{t}}=\frac{1}{k_{2}{q_{e}}^{2}}+\;\frac{1}{q_{e}}.t$	$\frac{t}{q_{t}}$ vs. *t*
Intra-particle diffusion kinetic model	$q_{t}=k_{p}.t^{0.5}+c$	*q_t_* vs. *t*^0.5^

**Table 2:** Kinetic parameters and correlation coefficient for Cd^2+^ adsorption by garbage ash.

*C_e_*	*q_e_*, exp [mg/g]	Pseudo first order			Pseudo second order			Intraparticle diffusion	
		*q_e_* (mg/g)	*K*_1_ (min^–1^)	$R^{2}$	*q_e_* (mg/g)	*K*_2_ (min^−1^)	$R^{2}$	*K_p_* [mg/g min^−0.5^]	*R*^2^
20	40	28.77	−0.010	0.94	36.33	0.002	0.96	5.47	0.95
50	80	41.28	−0.003	0.91	47.19	0.010	0.99	1.35	0.94
100	128	9.72	−0.020	0.81	128.37	0.015	1	1.73	0.65
150	221	11.38	−0.020	0.94	220.89	0.012	0.99	1.26	0.96

**Table 3:** Adsorption isotherms and obtained parameters for Cd^2+^ removal by garbage ash.

Isotherm	Linear form	Plot	Parameter	
Langmuir	$\frac{\mathrm{Ce}}{\mathrm{qe}}=\frac{1}{\mathrm{qm}}\mathrm{Ce}+\frac{1}{\mathrm{qmb}}$	$\frac{C_{e}}{q_{e}}\mathrm{vs}.\;C_{e}$	*q*_max_ (mg/g)	100.2490
			*K_L_* (L/mg)	0.5031
			*R*^2^	0.9975
Freundlich	$\mathrm{Log}q_{e}=\log\;K_{F}+\frac{1}{n}\;\log\;C_{e}$	$\log q_{e}\mathrm{vs}.\;\log C_{e}$	*K_F_*(mg/g(L/mg)^1/n^)	52.1889
			*n*	6.7140
			*R*^2^	0.9729
Temkin	$q_{e}=B\ln.A+B\ln C_{e}$	$q_{e}\mathrm{vs}.\;\ln C_{e}$	A (L/mg)	0.1701
			B	9.5318
			*R*^2^	0.9908

**Table 4:** Thermodynamic parameters for Cd^2+^ removal by garbage ash.

Temperature (K)	$\Delta G{}^{\circ}({\mathrm{kJ}\;\;\mathrm{mol}}^{-1})$	$\Delta H{}^{\circ}({\mathrm{kJ}\;\mathrm{mol}}^{-1})$	$\Delta S{}^{\circ}({\mathrm{kJ}\;\;\mathrm{mol}}^{-1}{\;K}^{-1})$
298	−0.183	21.206	7.391
308	−2.251		
318	−2.813		
328	−2.221		

## 2 Experimental design, materials and methods

### 2.1 Preparation of garbage ash

The sampling of garbage was performed according to physical and chemical sampling methodology proposed by the Iranian National Standard Organization. The waste samples were collected from the garbage separated for composting in Mashhad solid waste management organization located in Mashhad, Iran. In order to prepare the adsorbent, the samples were placed in oven to remove any moisture. For the preparation of ash, the sample was placed in a furnace at 550 °C for 4.5 h and was kept in desiccator after cooling.

### 2.2 Experimental procedures

Adsorption of Cd^2+^ from synthetic aqueous solution using garbage ash was performed in batch experiments. A stock solution of Cd^2+^ with a concentration 1000 ppm was prepared by dissolving appropriate quantity of Cd(NO_3_)_2_ in 1 L of deionized water. The required concentrations of Cd^2+^ solution were prepared by dilution of stock solution. The pH of solution was adjusted by 0.1 M HCl or 0.1 M NaOH. The Cd^2+^ solution containing different adsorbent dosages were placed in shaker incubator at 150 rpm at various time intervals. Finally, samples were filtered through Whatman papers No. 0.45 µm and the residual concentrations of Cd^2+^ were analyzed by an Atomic Absorption Spectrophotometer (AAS).The effect of key variables, such as initial cadmium (II) concentration (20, 50, 100, 150, 200 mg/L), contact time (2, 5, 15, 30, 45, 60 min), adsorbent dose (0.2, 0.5, 1, 2, 3 gr/L), pH (2–12) and temperature (20, 30, 40, 50 °C) were investigated.The experiments were conducted in duplicate and the results were reported as averages. The removal efficiency of Cd^2+^ ion (%*R*) and the adsorption capacity qe(mg/g) of the Cd^2+^ ion adsorbed per unit mass of adsorbent was calculated by the following equation [1]:

(1) $$\%R=\frac{C_{0}-C_{e}}{C_{0}}\times 100$$

(2) $$q_{e}=\;\frac{{(C}_{0}-C_{e})V}{m}$$

where, *C_0_* and *C_e_* is the initial concentration of Cd^2+^ and the equilibrium concentration of Cd^2+^ in solution in mg/L, respectively, *V* is the volume of the solution in *L*, and *m* is mass of the garbage ash in g.

### 2.3 Kinetic modeling

The experimental data were analyzed using kinetic models like pseudo firstorder, pseudo secondorder and intraparticle diffusion [2]. The kinetic equations are presented in Table 1. The kinetic study was performed by placing 0.5 g of adsorbent dosage in 1 L solution in concentration range 20–150 mg/L at an optimum pH of 6 under varying time intervals (5–60 min) at 25 °C and 150 rpm. In this equation, *q_e_* and *q_t_* is the adsorption capacity of Cd^2+^(mg/g) at equilibrium and at time *t*, respectively; *k*_1_ (min^−1^) is the rate constant of pseudo firstorder which can be computed from the slope of the linear plot of log (*q_e_*−*q_t_*) versus time, $k_{2}$ (min^−^^1^) is the pseudo second order rate constant. Slope of the plot of *t*/*q_t_* against *t* yield *k*_2_ value. In the intraparticle diffusion model, *K_p_* and *C* is the intraparticle diffusion constant and intercept, respectively. The value of *K_p_* was calculated from slope of the plot of *q_t_* against *t*^0.5^ [3], [4], [5].

### 2.4 Isotherm modeling

In order to describe the adsorption mechanism of Cd^2+^on the garbage ash, isothermal studies were used. The obtained data were evaluated using the isotherm models including the Langmuir, Freundlich and Temkin [6]. Batch adsorption isotherm tests were carried out at different initial concentrations from 20 to 200 mg/L under optimized conditions at pH around six and temperature of 25 °C.The linear forms of the isotherm equations are given in Table 2. According to isotherm equations, *C_e_* and *q_e_* is the equilibrium concentration of Cd^2+^ (mg/L) and the amount of Cd^2+^ adsorbed per unit weight of adsorbents at equilibrium (mg/g),respectively. *q_m_* is the maximum adsorption capacity for the Langmuir isotherm (mg/g), $K_{L}$ is the Langmuir isotherm constant (L/mg). *K_F_* and *n* is Freundlich adsorption constants related to adsorption capacity and adsorption intensity, respectively, and were determined from slope and intercept of the plot of ln (*q_e_*) versus ln (*C_e_*). In Temkin equation, A and B are the binding constant (L/mg) and constant corresponding to the heat of adsorption [7], [8], [9], [10].

### 2.5 Thermodynamic modeling

Thermodynamic parameters of the adsorption process such as enthalpy change (ΔH°), entropy change (ΔS°) and Gibbs free energy change (ΔG°) at temperatures 20, 30, 40 and 50 °C were estimated using the following equations:

(3) $$\Delta G^{{}^{\circ}}=-{\mathrm{RTlnK}}_{L}$$

(4) $${\mathrm{LnK}}_{L}=\;\frac{\Delta S{}^{\circ}}{R}-\frac{\Delta H{}^{\circ}}{\mathrm{RT}}$$

where, ΔG° is Gibbs free energy change (J/mol), ΔS° is entropy change (J/mol K), ΔH° is enthalpy change (J/mol), R is the ideal universal gas constant (8.314 J/K mol), and *T* is the temperature (Kelvin). (ΔH°) and (ΔS°) is determined using the plot of ln *K_L_* versus 1/*T* [11], [12].

### 2.6 The effects of coexisting ions

In order to determine the effects of cations including Fe^2+^, Mn^2+^, Pb^+^, Ca^2+^, Mg^2+^, Na^+^ and K^+^ on the removal of Cd^2+^ by garbage ash, FeCl_3_, MnSO_4_, Pb(NO_3_)_2_, CaCl_2_, MgCl_2_, NaCl and KCl salts were used.
